# mTOR inhibition impacts the flagellin-augmented inflammatory and antimicrobial response of human airway epithelial cells to *Pseudomonas aeruginosa*

**DOI:** 10.1371/journal.pone.0321462

**Published:** 2025-05-08

**Authors:** Christine C.A. van Linge, Katina D. Hulme, Hessel Peters-Sengers, Robert F.J. Kullberg, Menno D. de Jong, Colin A. Russell, Alex F. de Vos, Tom van der Poll

**Affiliations:** 1 Center for Infection and Molecular Medicine, Amsterdam University Medical Center, University of Amsterdam, Amsterdam, The Netherlands; 2 Amsterdam Institute for Immunology and Infectious Diseases, Amsterdam, The Netherlands; 3 Department of Medical Microbiology and Infection Prevention, Amsterdam University Medical Center, University of Amsterdam, Amsterdam, The Netherlands; 4 Department of Global Health, School of Public Health, Boston University, Boston, Massachusetts, United States of America; 5 Division of Infectious Diseases, Amsterdam University Medical Center, University of Amsterdam, Amsterdam, The Netherlands; Universidad Nacional de la Plata, ARGENTINA

## Abstract

**Objective:**

The airway epithelium provides a first line of defense against pathogens by release of antimicrobial factors and neutrophil-attracting chemokines. *Pseudomonas (P.) aeruginosa*, a Gram-negative bacterium that expresses flagellin as an important virulence factor, is a common cause of injurious airway inflammation. The aim of our study was to determine the contribution of flagellin to the inflammatory, antimicrobial, and metabolic responses of the airway epithelium to *P. aeruginosa*. Furthermore, as we previously showed that targeting mTOR limited the glycolytic and inflammatory response induced by flagellin, we assessed the effect of rapamycin on human bronchial epithelial (HBE) cells stimulated with flagellated and non-flagellated *P. aeruginosa.*

**Methods:**

Primary pseudostratified HBE cells, cultured on an air-liquid-interface, were treated on the basolateral side with medium, vehicle or rapamycin, exposed on the apical side with flagellated or flagellin-deficient *P. aeruginosa*, and analyzed for their inflammatory, antimicrobial, and glycolytic responses.

**Results:**

Flagellin augmented the *P. aeruginosa*-induced expression of antimicrobial factors and secretion of chemokines by HBE cells but did not further increase the glycolytic response. Treatment of HBE cells with rapamycin inhibited mTOR activation in general and flagellin-augmented mTOR activation in particular, but did not affect the glycolytic response. Rapamycin, however, diminished the flagellin-augmented inflammatory and antimicrobial response induced by *Pseudomonas*.

**Conclusions:**

These results demonstrate that flagellin is a significant factor that augments the inflammatory and antimicrobial response of human airway epithelial cells upon exposure to *P. aeruginosa* and suggest that mTOR inhibition by rapamycin in the airway epithelium diminishes these exaggerated responses.

## 1. Introduction

*Pseudomonas (P.) aeruginosa* is a flagellated Gram-negative bacterium and one of the most frequent causative pathogens in pneumonia [1]. Patients at risk include those who are hospitalized and/or suffer from chronic lung disorders, such as cystic fibrosis or chronic obstructive pulmonary disease, which are associated with airway colonization by *Pseudomonas* [1]. Furthermore, *P. aeruginosa* was defined as one of the six leading pathogens causing antimicrobial resistance-associated deaths worldwide [2], emphasizing the importance of developing alternative therapies.

The respiratory epithelium in the lung is responsible for the first line of defense against inhaled pathogens and can be activated through different pattern recognition receptors, such as Toll-like receptors (TLRs) [3–5]. TLR2, TLR4, and TLR5 are involved in the recognition of *P. aeruginosa* in the lung, as exemplified by reduced host defense against this pathogen in mice deficient for these TLRs or the common adaptor myeloid differentiation primary response-88 [6–9]. TLRs can be activated by specific *Pseudomonas*-derived molecular patterns, including lipoproteins (TLR2), lipopolysaccharide (LPS; TLR4) and flagellin (a structural component of the flagellum; TLR5) [9–12]. Previously, we and others have shown that primary human bronchial epithelial (HBE) cells can be activated by flagellin and by *P. aeruginosa* [11–15], leading to the expression and secretion of neutrophil-attracting chemokines and antimicrobial proteins [12,14,15]. However, the contribution of flagellin to the *Pseudomonas-*induced epithelial response has not yet been described in HBE cells.

Glycolysis is a crucial metabolic pathway that converts glucose into pyruvate and lactate, providing cells with energy for the production and release of inflammatory cytokines. Mechanistic target of rapamycin (mTOR) plays a central role in regulating cellular metabolism and stimulates glycolysis [16,17]. Recently, we found that inhibition of mTOR activation by rapamycin, a drug prescribed for various chronic diseases and prevention of transplant rejection [18], impeded glycolysis and limited the secretion of chemokines by flagellin-stimulated human airway epithelial cells [14]. Yet, whether mTOR inhibition by rapamycin could reduce inflammation during *P. aeruginosa* infection is currently unknown.

The current study had two main objectives: first, to determine the contribution of flagellin to the *P. aeruginosa*-induced inflammatory and antimicrobial response by primary HBE cells, and second, to establish whether targeting of mTOR by rapamycin can modulate the inflammatory and antimicrobial responses of the human airway epithelium induced by flagellated and non-flagellated *Pseudomonas.* For this purpose, we studied primary HBE cells, cultured in a pseudostratified manner on an air-liquid-interface, after treatment on the basolateral side with rapamycin and exposure at the apical side with flagellated *P. aeruginosa* (PAK) or flagellin-deficient (PAKΔF) bacteria. The results of our study indicate that flagellin augments the *P. aeruginosa*-induced expression of chemokines and antimicrobial factors, and that these responses are diminished by inhibition of mTOR activation.

## 2. Materials and methods

### 2.1. Preparation of bacterial stocks

Wild-type *P. aeruginosa* (PAK) and flagellin-deficient PAK, generated by deletion of the fliC gene (PAK ∆ fliC), hereafter designated PAKΔF, were cultured as described [19,20]. Briefly, bacteria were grown to mid-logarithmic phase in Luria broth at 37°C with shaking, washed with 0.9% NaCl, and resuspended in phosphate-buffered saline (PBS). Bacterial numbers were determined by plating serial dilutions on blood agar plates. Bacteria were heat-killed for 20 minutes at 70°C, stored at 4°C, and used the next day. We previously showed that heat treatment did not affect the stimulatory capacity of flagellin [21].

### 2.2. HBE cell culture and stimulation

Primary human bronchial epithelial (HBE) cells were cultured as previously described [12]. Briefly, primary bronchial epithelial cells were isolated from healthy lung tissue obtained anonymously from patients undergoing a lobectomy for lung cancer (Amsterdam UMC approved study protocol 2015–122#A2301550). Written informed consent was obtained before surgery and tissue was sampled between July 1^st^ 2015 to December 30^th^ of 2018. Primary epithelial cells were isolated and differentiated following Fulcher’s protocol [22]. Passage 2–4 primary HBE cells were viably frozen between 2015 and 2018. Cells were thawed for the current study. At this point, cells were differentiated on Transwell inserts (Corning) [22]. When the cells on the inserts reached confluency, an air-liquid interface was formed with PneumaCult-ALI medium (StemCell Technologies) on the basolateral side, and removal of culture medium from the apical side. For bacterial stimulation, HBE cells were inoculated on the apical side with 4*10^6^ colony-forming units of PAK or PAKΔF per insert in a volume of 20 μl (or 20 μl PBS as control) and incubated at 37°C. After 24 hours, the apical surface was washed with 200 μl PBS. Basolateral medium and apical wash were stored at -20°C until further analysis. HBE cells were stored in RNA lysis/binding buffer at -80°C for RNA isolation. For inhibition of mTOR, HBE cells were treated with 10 nM rapamycin (Cayman chemical) in the basolateral culture medium 1 hour prior to bacterial stimulation, as described [14].

### 2.3. Cytokine and lactate measurements

Chemokine CXC motif ligand (CXCL)1, CXCL8, CC chemokine ligand (CCL)20, granulocyte-colony stimulating factor (G-CSF), and calprotectin were measured by ELISA according to the manufacturer’s instructions (R&D Systems). Lactate was measured as described [14] using an assay in which lactate was oxidized and the resulting hydrogen peroxide was coupled to the conversion of Amplex Red reagent (Thermo Fisher Scientific) to fluorescent resorufin by horseradish peroxidase.

### 2.4. mRNA analysis

Total mRNA was isolated from HBE cells using the High Pure RNA Isolation Kit (Roche), following the manufacturer’s instructions. cDNA synthesis and qPCR were performed as previously described [14]. Data were analyzed with LinRegPCR software based on PCR efficiency values derived from amplification curves. *HPRT* was used as a housekeeping gene for normalization. Primers for *HPRT*, *CXCL1*, *CXCL8*, *CCL20*, *S100A8,* and *S100A9* were similar as described [12]. All other primers are listed in Table 1.

**Table 1:** Primer sequences for qPCR analysis of *GCSF*, *HK2,* and *DEFB4.*

Gene	Forward	Reverse
*GCSF*	GATGGAAGAACTGGGAATGG	GACACCTCCAGGAAGCTCTG
*HK2*	GGGCATCTTTGAAACCAAGT	CCACAGTGCACACCTCCTTA
*DEFB4*	ATCTCCTCTTCTCGTTCCTCTTC	GGGCAAAAGACTGGATGACA

### 2.5. Immunoblot analysis

Western blot analysis was performed as previously described [14]. Briefly, HBE cells were lysed in RIPA buffer supplemented with HALT protease and phosphatase inhibitor (Thermo Fisher Scientific) and heat-denatured in reducing sample buffer (Laemmli buffer) prior to 12.5% polyacrylamide gel electrophoresis. Blots were first incubated with antibodies against β-Actin (4967L; Cell Signaling Technologies) and phosphor-S6 Ribosomal Protein (Ser235/236) (2211S; Cell Signaling Technologies) and subsequently with horse radish peroxidase-conjugated goat anti-rabbit IgG (7074S; Cell Signaling Technologies). Blots were incubated with the Lumi-Light detection kit (Roche) and pictures were taken using a ChemiDoc MP (Bio-Rad). Western blot results were quantified by densitometry analysis using Adobe Photoshop version 24.3.0 (Adobe).

### 2.6. Statistical analysis

Statistical analysis was performed using a mixed model with a random intercept on the level of experiment and donor, with 3–4 technical replicates from 2 donors. Mixed models were adjusted for donor by including it as a covariate. Outcomes were log-transformed for analyses. All (co)variances were assumed equal and equivalent to a compound symmetry covariance structure. If the homogeneity of variances across groups was violated, as determined by Levene’s test (p < 0.05), we used non-parametric Mann-Whitney U tests to compare groups that did not meet this assumption. In case the overall group difference was significant, determined by type-III Wald test in the mixed model analyses (p < 0.05), then each stimulation was compared with the reference category to identify differences between groups. We used package “nlme” (version 3.1–164) for mixed models in the R statistical framework. Single-donor data from the rapamycin experiment were analyzed using GraphPad Prism version 9 (GraphPad Software). To this end, parametric variables were analyzed using a 2-way analysis of variance (comparison between 3 or more groups) or a Student’s t-test (2-group comparison). Non-parametric variables were analyzed using Kruskall-Wallis and Mann-Whitney U tests. In graphpad, multiple comparisons were Benjamini-Hochberg-corrected for multiple testing. Statistical significance is shown as *p < 0.05, **p < 0.01, ***p < 0.001.

## 3. Results

### 3.1. Flagellin augments *P. aeruginosa*-induced chemokine release by HBE cells

**Fig 1:**
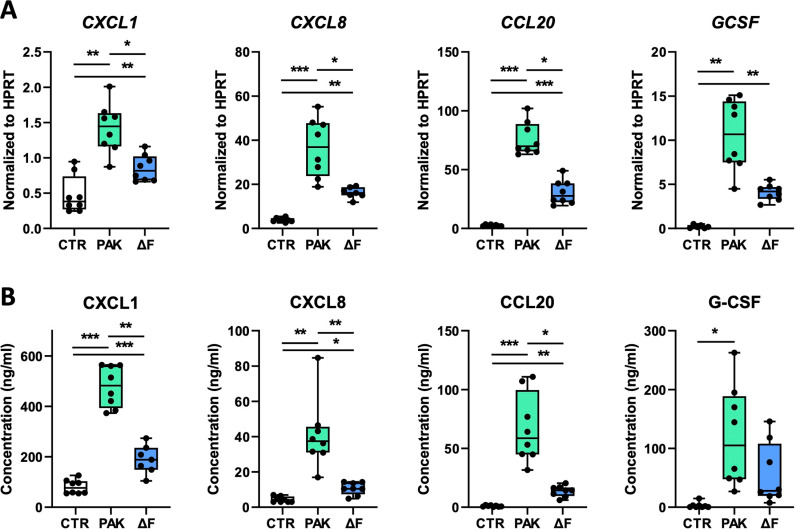
Flagellin augments *P. aeruginosa*-induced chemokine release by HBE cells. HBE cells were exposed at the apical side to vehicle (CTR), 4*10^6^ CFUs of flagellated *P. aeruginosa* (PAK) or flagellin-deficient PAK (ΔF) for 24 hours, at which point samples were collected. mRNA levels of *CXCL1*, *CXCL8*, *CCL20*, and *GCSF*, normalized to HPRT (A), CXCL1, CXCL8, CCL20 levels in the basolateral medium and G-CSF in the apical wash (B). Data are presented as box and whiskers from 2 donors with 4 technical replicates per group. *Indicates significance between the indicated groups. *p < 0.05; **p < 0.01; ***p < 0.001.

We showed earlier that both *P. aeruginosa* and flagellin induced an inflammatory response in pseudostratified HBE cells characterized by the secretion of CXCL1, CXCL8, CCL20 [12], and G-CSF [14]. To determine the contribution of flagellin to the *Pseudomonas-*induced epithelial response, we treated HBE cells on the apical side with either PAK, PAKΔF, or medium alone and measured the expression and secretion of inflammatory mediators 24 hours later. Both PAK and PAKΔF induced the expression of *CXCL1, CXCL8*, *CCL20,* and *GCSF* compared to medium control, yet PAK induced significantly higher mRNA levels compared to PAKΔF (Fig 1A). Similarly, both PAK and PAKΔF induced the secretion of CXCL1, CXCL8, and CCL20 in the basolateral medium, with higher chemokine concentrations after exposure to PAK compared to PAKΔF (Fig 1B). G-CSF levels in the apical wash were increased after stimulation with PAK and PAKΔF but reached significance only for PAK (Fig 1B). These findings indicate that the expression and secretion of chemokines by HBE cells in response to *Pseudomonas* is augmented by flagellin.

### 3.2. Flagellin increases the *P. aeruginosa*-induced expression of antibacterial defense genes by HBE cells

**Fig 2:**
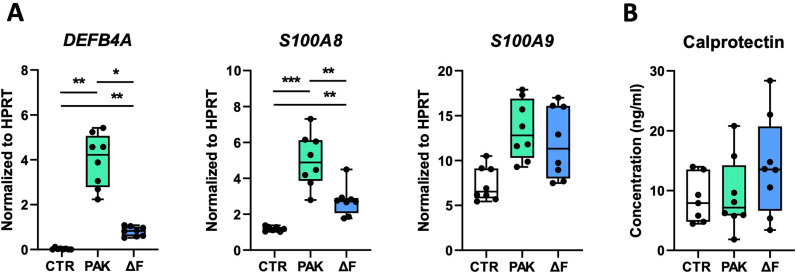
Flagellin increases the *P. aeruginosa*-induced expression of antibacterial defense genes by HBE cells. HBE cells were exposed to vehicle (CTR), flagellated (PAK) or flagellin-deficient (ΔF) *P. aeruginosa* as described in Fig 1. Relative mRNA levels of *DEFB4A*, *S100A8*, and *S100A9* after 24 hours (A), and protein levels of calprotectin in apical wash (B). Data are presented as box and whiskers from 2 donors with 4 technical replicates per group. *Indicates significance between the indicated groups. For *DEFB4A*, the homogeneity of variance could not be assumed, because of the minimal variance in the control group. Therefore, we compared the PAK and PAKΔF group separately. *p < 0.05; **p < 0.01; ***p < 0.001.

Upon encountering bacteria, the bronchial epithelium secretes antimicrobial peptides that aid in host defense by exerting direct antimicrobial effects and recruiting other immune cells to the site of infection [5,23]. We determined expression levels of genes encoding β-defensin 4a (*DEFB4A*) and calprotectin (*S100A8* and *S100A9*), factors involved in antibacterial defense, after 24 hours of exposure to PAK or PAKΔF. Both PAK and PAKΔF induced the expression of *DEFB4A* and *S100A8* compared to control, with significantly higher levels after PAK stimulation compared to the flagellin-deficient strain (Fig 2A). In contrast to increased expression of *S100A8*, no differences were found in calprotectin protein levels in apical wash upon exposure to PAK or PAKΔF (Fig 2B). These data suggest that flagellin augments the PAK-induced expression of antibacterial defense genes.

### 3.3. Exposure to *P. aeruginosa* induces a glycolytic response in HBE cells

**Fig 3:**
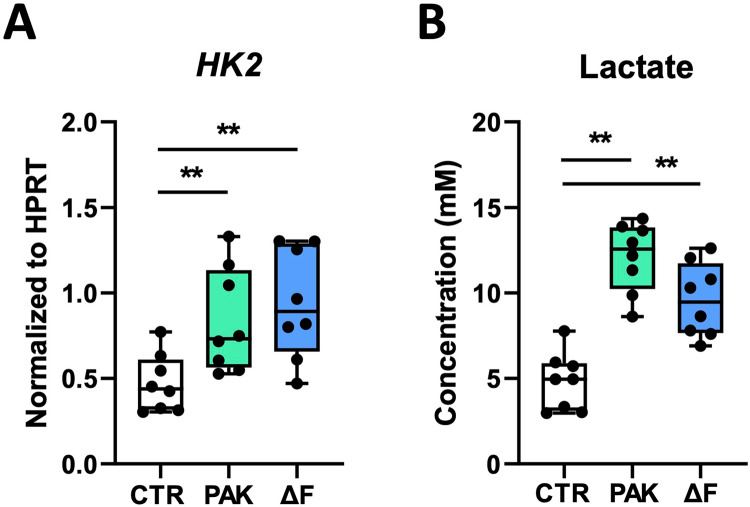
Exposure to *P. aeruginosa* induces a glycolytic response in HBE cells. HBE cells were exposed to vehicle (CTR), flagellated (PAK) or flagellin-deficient (ΔF) *P. aeruginosa* as described in Fig 1. Relative mRNA levels of *HK2* (**A**) and lactate levels in basolateral medium (**B**) after 24 hours stimulation. Data are presented as box and whiskers from two donors with 4 technical replicates per group. *Indicates significance between the indicated groups. **p < 0.01.

Previously, we reported that purified flagellin triggered glycolysis in HBE cells after 24-hour stimulation [14]. To determine whether flagellin impacted the glycolytic response of HBE cells evoked by *P. aeruginosa*, we measured the mRNA expression of hexokinase-2 (*HK2),* the rate-limiting enzyme required for the first step in glycolysis [14], in HBE cells and the release of lactate in the basolateral medium after exposure to PAK or PAKΔF. Both PAK and PAKΔF increased mRNA levels of *HK2* (Fig 3A) and the release of lactate compared to unstimulated cells (Fig 3B). Flagellin, however, did not affect the glycolytic response of HBE cells as *HK2* mRNA levels and lactate concentrations were not different after exposure to PAK or PAKΔF. These results suggest that *P. aeruginosa* induces an increase in glycolytic activity in the human airway epithelium independent of flagellin.

### 3.4. Rapamycin inhibits *P. aeruginosa*-induced mTOR activation in HBE cells but has little impact on lactate release

**Fig 4:**
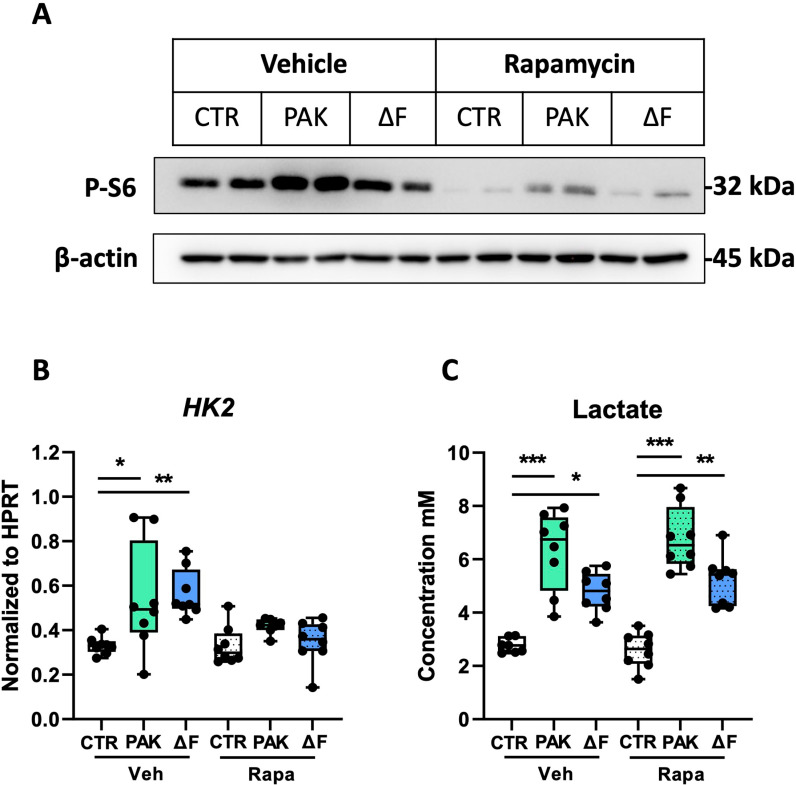
Rapamycin inhibits *P. aeruginosa*-induced mTOR activation in HBE cells but has little impact on lactate release. HBE cells were treated with vehicle (Veh) or rapamycin (Rapa) and, after 1 hour, exposed to vehicle (CTR), flagellated (PAK), or flagellin-deficient (ΔF) *P. aeruginosa*. Western blot of phosphorylated ribosomal protein S6, a downstream target of mTOR, and β-actin as a control after 2 hours of stimulation (A). Ratio of mean intensity of pS6 and β-actin bands determined by densitometry (B). Relative mRNA levels of *HK2* (C), and lactate levels in basolateral medium (**D**) 24 hours after exposure. Data are presented as box and whiskers from one donor with 8 technical replicates per group. *Indicates significance between the indicated groups. *p < 0.05; **p < 0.01; ***p < 0.001.

Since mTOR is a central regulator of cellular metabolism in flagellin-treated HBE cells and targeting of mTOR by rapamycin inhibited the flagellin-induced glycolytic and inflammatory response after 24-hour stimulation [14], we next assessed the effect of mTOR inhibition on the response of HBE cells to *P. aeruginosa*. To this end, we treated HBE cells with rapamycin before exposure to PAK or PAKΔF and first analyzed the phosphorylation of ribosomal protein S6, a direct downstream target of mTOR [18], by Western blot analysis (Fig 4A). PAK, in contrast to PAKΔF, tended to increase the expression of phospho-S6 in HBE cells (Fig 4B). Treatment with rapamycin significantly inhibited the activation of the mTOR pathway, as revealed by lower levels of phospho-S6 in HBE cells exposed to medium, PAK, or PAKΔF. Next, we determined whether rapamycin also decreased the glycolytic response upon stimulation with *P. aeruginosa* by analysis of *HK2* expression and lactate release. Rapamycin did not impact the expression of *HK2* or the release of lactate, compared to vehicle, without bacterial stimulation. Upon exposure to PAK or PAKΔF, rapamycin impeded the increased expression of *HK2* (Fig 4C). Strikingly, however, increased lactate levels in the supernatant of HBE cells after stimulation with PAK and PAKΔF were not affected by rapamycin treatment (Fig 4D). These data indicate that rapamycin inhibits the augmented mTOR activation in HBE cells exposed to flagellated *P. aeruginosa*, but does not affect their glycolytic flux as revealed by lactate release.

### 3.5. Inhibition of mTOR activation diminishes the inflammatory and antimicrobial response induced by flagellated *P. aeruginosa*

**Fig 5:**
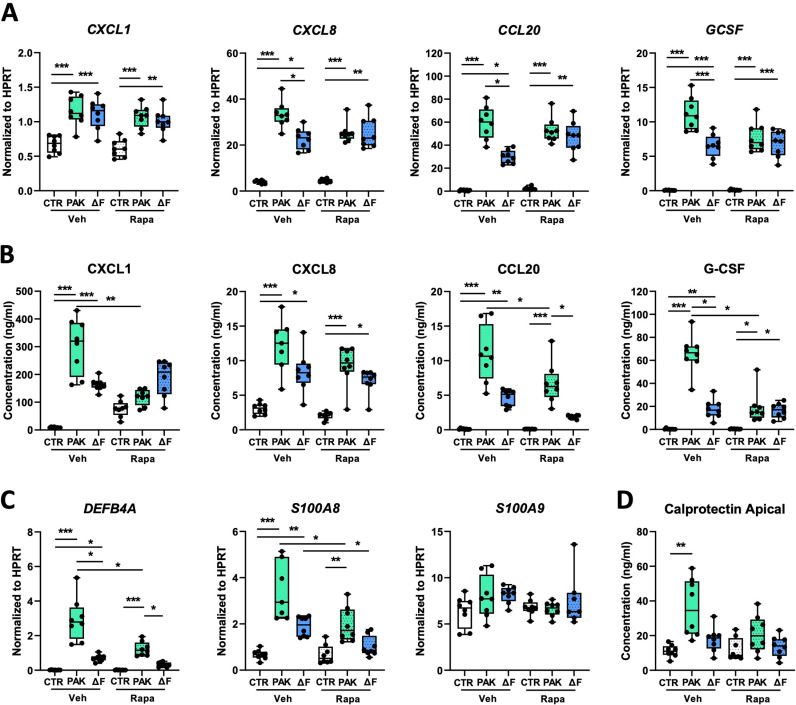
Inhibition of mTOR activation diminishes the inflammatory and antimicrobial response induced by flagellated *P. aeruginosa.* HBE cells were treated with vehicle (Veh) or rapamycin (Rapa) and, after 1 hour, exposed to vehicle (CTR), flagellated (PAK), or flagellin-deficient (ΔF) *P. aeruginosa* for 24 hours, at which point samples were collected. Relative mRNA levels of *CXCL1, CXCL8, CCL20*, and *GCSF* (A), chemokine levels in the basolateral medium and G-CSF in the apical wash (B), relative mRNA levels of *DEFB4A*, *S100A8 and S100A9* (C), and calprotectin levels in apical wash (D). Data are presented as box and whiskers from one donor with 8 technical replicates per group. *Indicates significance between the indicated groups. *p < 0.05; **p < 0.01; ***p < 0.001.

After having established that rapamycin inhibits mTOR activation in *Pseudomonas*-exposed HBE cells, we determined the effect of rapamycin on the PAK- and PAKΔF-induced inflammatory response of HBE cells. Rapamycin inhibited the flagellin-augmented expression of *CXCL8*, *CCL20*, and *GCSF*, but not of *CXCL1* (Fig 5A). After rapamycin treatment, PAK and PAKΔF induced similar mRNA levels of these genes in HBE cells. In addition, rapamycin reduced the secretion of CXCL1, CCL20, and G-CSF upon exposure to PAK (not CXCL8 secretion), but did not impact PAKΔF-induced secretion of these mediators, except for CCL20 (Fig 5B). Next, we determined the effect of rapamycin on the PAK- and PAKΔF-induced antimicrobial response of HBE cells. Rapamycin decreased the flagellin-augmented mRNA levels of *DEFB4A* and *S100A8* upon exposure to *Pseudomonas*, as well as *S100A8* mRNA levels induced by PAKΔF exposure (Fig 5C). The flagellin-augmented secretion of calprotectin was diminished in rapamycin-treated HBE cells (Fig 5D). Taken together, these data indicate that mTOR inhibition by rapamycin diminishes both the flagellin-augmented inflammatory and antimicrobial response induced by *P. aeruginosa*.

## 4. Discussion

Flagellin has been implicated as a potent activator of the pulmonary epithelium by inducing the secretion of immune cell-attracting chemokines and antimicrobial proteins [11]. In the present study, we investigated the contribution of flagellin to the inflammatory and antimicrobial response of pseudostratified HBE cells in the context of *Pseudomonas* infection. We demonstrate that flagellin potentiated chemokine secretion and to some extent also the expression of antimicrobial proteins and metabolic response evoked by *P. aeruginosa* in the human airway epithelium. Moreover, our study reveals that mTOR inhibition by rapamycin diminished this flagellin-augmented inflammation to the level evoked by non-flagellated *P. aeruginosa*. These findings indicate that the flagellin-induced airway epithelial response to *P. aeruginosa* is regulated through mTOR.

Our findings that flagellated *P. aeruginosa* induced higher expression and secretion of chemokines by HBE cells compared to flagellin-deficient *P. aeruginosa* are in line with earlier studies by our group using HBE cells and purified flagellin [12,14,15], and findings of others showing that soluble bacterial ligands derived from flagellated *P. aeruginosa*, but not of flagellin-deficient *P. aeruginosa,* increased the release of CXCL8 by HBE cells after 24-hour stimulation [24]. Furthermore, a study with primary pseudostratified tracheal epithelial cells on an air-liquid interface also showed that flagellated *P. aeruginosa* increased the release of CXCL8 to the basolateral medium compared to a flagellin-deficient strain [25]. These findings support the notion that flagellin is a potent activator of inflammatory responses by lung epithelial cells during *Pseudomonas* infection in the lung.

The expression of TLR5 by airway epithelial cells is regarded as crucial for host defense against *P. aeruginosa* [26–28], both for induction of antimicrobial factors and the release of cytokines and chemokines triggering the attraction and activation of immune cells including neutrophils. Analysis of TLR5 mRNA levels in HBE cells revealed that neither PAK, PAK ∆ F or rapamycin impacted on its expression (data not shown). Our finding that PAK lacking the TLR5 ligand flagellin triggered a substantial inflammatory response in HBE cells, however, may be explained by activation of pattern recognition receptors via other ligands of *Pseudomonas*. Previously, we demonstrated that HBE cells, besides TLR5, express other TLRs including TLR1, TLR2, and TLR3 [12]. However, HBE cells do not express TLR4 and are unresponsive to lipopolysaccharide (LPS) [14]. Both PAK and PAKΔF have been found to activate TLR2 [13], and several components of *P. aeruginosa* have been identified as TLR2 ligands [9,29,30]. In agreement, lung epithelial cells derived from mice deficient in both TLR2 and TLR4 displayed severely hampered chemokine release upon PAKΔF exposure[6,31]. Thus, although the inflammatory response of HBE cells upon exposure to *P. aeruginosa* is vastly augmented by flagellin-mediated triggering of TLR5, other receptors also contribute to the *P. aeruginosa*-induced activation.

In line with our earlier study with flagellin-stimulated HBE cells [14], we found that *P. aeruginosa* induces a glycolytic response in the airway epithelium, as revealed by increased levels of hexokinase 2 mRNA and secreted lactate. Flagellin expression by *Pseudomonas*, however, did not impact this response, indicating that ligands other than flagellin induce the glycolytic response of HBE cells in the context of *P. aeruginosa* exposure. Also consistent with our former study investigating the effect of purified flagellin [14], was the finding that PAK, but not PAKΔF, triggered the activation of the mTOR pathway in HBE cells, as indicated by increased phospho-S6 protein levels. These findings corroborate a recent study showing that *P. aeruginosa* upregulated the expression of genes involved in mTOR signaling in human airway organoids [32]. Strikingly, we found that rapamycin, although reducing *HK2* mRNA levels, did not diminish lactate secretion by HBE cells upon exposure to *P. aeruginosa*, opposite from our previous result that mTOR inhibition impeded glycolysis after 24-hour exposure to flagellin [14]. Nevertheless, consistent with the effect of rapamycin on flagellin-stimulated HBE cells [14], rapamycin diminished the expression of inflammatory and antimicrobial factors upon exposure to PAK. One study investigated the effect of mTOR-inhibition on the inflammatory response of a mouse macrophage cell line and found that rapamycin decreased flagellin-induced cytokine production by preventing the activation of p65 and STAT3, downstream of the TLR5-mTOR signaling pathway [33]. These latter findings suggest that mTOR inhibition can directly prevent the inflammatory response induced by flagellin, regardless of glycolytic activation.

The effect of rapamycin on lung inflammation and bacterial infection in experimental models *in vivo* has been variable. We previously showed that rapamycin treatment reduced pulmonary chemokine production during flagellin-induced lung inflammation in mice [14]. Others showed that rapamycin treatment in mice inhibited LPS-induced lung inflammation, but augmented lung injury [34,35] Rapamycin treatment ameliorated lung inflammation and host defense against pulmonary *Burkholderia cenocepacia* infection in a mouse model of cystic fibrosis [36]. Rapamycin, however, did not impact host defense against pulmonary *Streptococcus pneumoniae* infection and exacerbated *Staphylococcus aureus* pneumonia [37,38]. These studies highlight the varying effects of rapamycin on the host response in the lung evoked by different bacteria and bacterial ligands and warrant further studies on rapamycin in *P. aeruginosa* pneumonia.

Our study has several limitations. First, we used heat-killed instead of live bacteria since our primary focus was on the effect of flagellin. Since motility of PAK may contribute to virulence by enhancing invasion of epithelial cells and by translocation across epithelial barriers, further studies with live PAK and PAK ∆ F, as well as other motility mutants [39] and complementation of PAK ∆ F with purified flagellin may clarify whether detection of flagellin or motility influences the response of HBE cells. Second, our study did not control for cell death. While differences in cytokine levels between PAK- and PAK ∆ F-stimulated cells may be explained by differing rates of cell death, we regard this an unlikely explanation since bacteria were heat-killed, PAK ∆ F lack an important virulence factor and the absence of flagellin did not impact on the expression and secretion levels of several other inflammation markers. Third, since we used primary bronchial epithelial cells from two different donors, inter-donor differences could partly explain why similar experiments did not yield identical results. Moreover, slight differences in the preparation of heat-killed bacterial stocks and the pseudostratified HBE cell layer, consisting of multiple layers of different cells [4], could to some extent also impact the results. Although we consider our model with pseudostratified bronchial epithelial cells cultured in an air-liquid interface as highly relevant for the human lung, further studies are required to determine whether our findings with rapamycin also apply to other cell types in the lung [3,40]. Alveolar epithelial type II (AECII) cells are capable of secreting a variety of inflammatory mediators and antimicrobial factors [41,42], and primary mouse AECII are responsive to *Pseudomonas* [43] and susceptible to rapamycin [44]. Human AECII, however, are hard to culture [4]. We did not consider the alveolar epithelial cell line A549 for our experiments, despite the expression of TLR5 and other pattern recognition receptors [45], since these cells do not develop apical-basal polarity and given their aberrant cellular metabolism [40] resulting from their malignant origin. Even more interesting would be to add alveolar macrophages and neutrophils to these models, as these immune cells are known to contribute to host defense against *Pseudomonas* [28,46] and are known to be responsive to rapamycin [3,47,48].

In summary, we demonstrate here that flagellin augments the inflammatory and antimicrobial responses of HBE cells upon exposure to *P. aeruginosa* and that mTOR inhibition diminishes these exaggerated responses. These results imply that targeting mTOR in airway epithelial cells may be beneficial for the treatment of superfluous lung inflammation during *Pseudomonas* infection.

## Supporting information

S1 File Western blots regarding Fig 4. (PDF)

S2 File Datasheet regarding Figs 1–5. (XLSX)
