# Supplementary figures and images for: mTOR inhibition impacts the flagellin-augmented inflammatory and antimicrobial response of human airway epithelial cells to Pseudomonas aeruginosa
Source: PLoS One. 2025 May 8;20(5):e0321462. doi: 10.1371/journal.pone.0321462 (PMC12061179; doi:10.1371/journal.pone.0321462)

# Western blot analysis

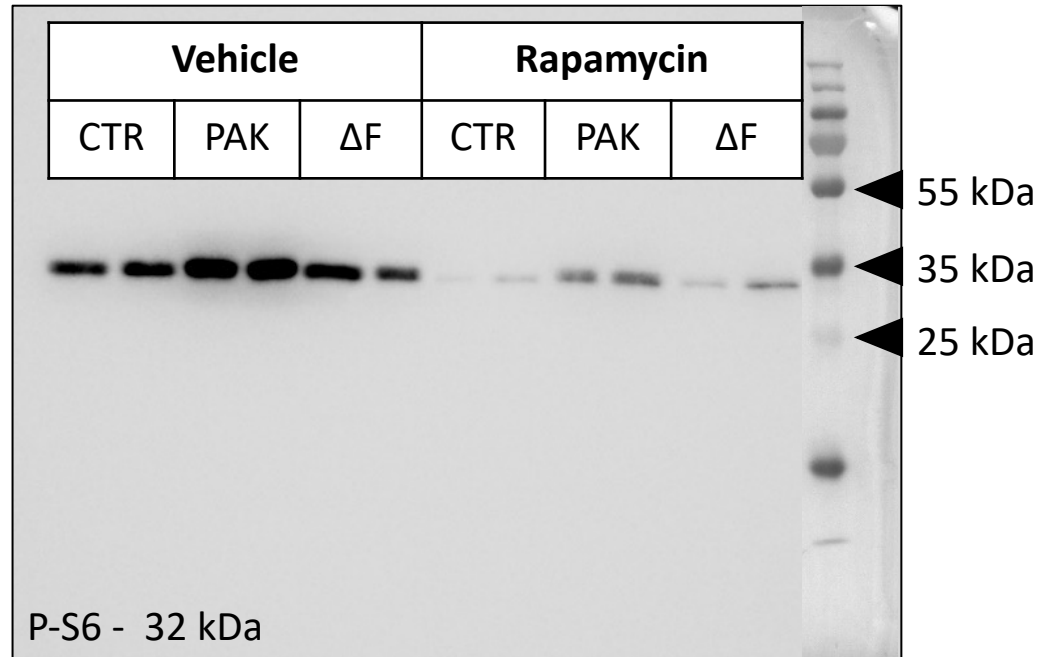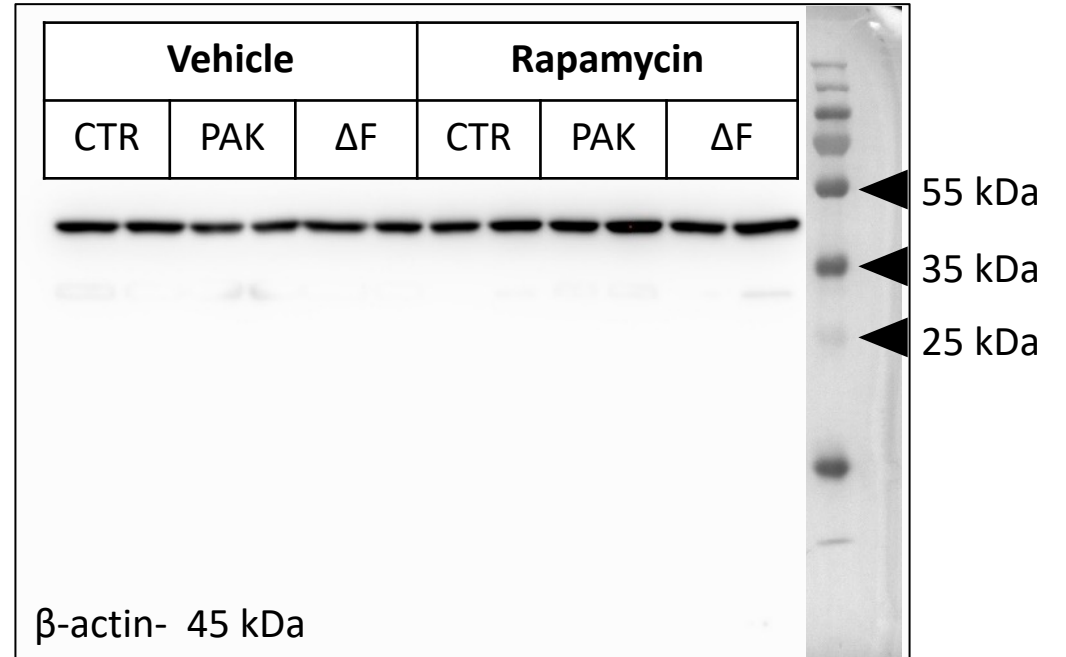

Supplement: S1 File — (PDF) [file pone.0321462.s001.pdf]
